# Integrated expression profiles analysis reveals novel predictive biomarker in pancreatic ductal adenocarcinoma

**DOI:** 10.18632/oncotarget.16732

**Published:** 2017-03-31

**Authors:** Hongzhe Li, Xinjing Wang, Yuan Fang, Zhen Huo, Xiongxiong Lu, Xi Zhan, Xiaxin Deng, Chenghong Peng, Baiyong Shen

**Affiliations:** ^1^ Department of Surgery, Ruijin Hospital, Shanghai Jiao Tong University School of Medicine, Shanghai, P.R. China; ^2^ Research Institute of Digestive Surgery, Ruijin Hospital, Shanghai Jiao Tong University School of Medicine, Shanghai, P.R. China; ^3^ Pancreatic Disease Centre, Ruijin Hospital, School of Medicine, Shanghai Jiao Tong University, Shanghai, P.R. China

**Keywords:** pancreatic ductal adenocarcinoma, microarray databases, prognosis, cell-matrix adhesion, biomarker

## Abstract

Pancreatic ductal adenocarcinoma (PDAC) is the most lethal human malignant tumor, with a dismal 5-year survival rate of less than 5%. The lack of specific symptoms at early tumor stages and the paucity of biomarkers contribute to the poor diagnosis of pancreatic ductal adenocarcinoma. To improve prognosis, a screening biomarker for early diagnosis of pancreatic cancer is in urgent need. We searched the databases of expression profiling by array on GEO, aiming at comparing gene expression profile of matched pairs of pancreatic tumor and adjacent non-tumor tissues, and we screen out 4 suitable series of gene expression microarray data (“GSE15471”, “GSE18670”, “GSE28735” and “GSE58561”). After carefully analyzing, 13 DEGs (MYOF, SLC6A6, S100P, HK2, IFI44L, OSBPL3, IGF2BP3, PDK4, IL1R2, ERO1A, EGLN3, PLAC8 and ACSL5) are significantly differentially expressed in four microarray databases in common. After analyzing mRNA expression data and clinical follow-up survey provided in the TCGA database and clinicopathological data of 137 pancreatic ductal adenocarcinoma patients, we carefully demonstrated that three of these differentially expressed genes (ERO1A, OSBPL3 and IFI44L) are correlated with poor prognosis of pancreatic ductal adenocarcinoma patients. In addition, we revealed that cell–matrix adhesion and extracellular matrix were top significantly regulated pathways in pancreatic ductal adenocarcinoma and depicted two protein-protein interactions networks of extracellular matrix related Genes which are dysregulated according to 4 gene expression microarray data mentioned above (“GSE15471”, “GSE18670”, “GSE28735” and “GSE58561”), hoping to shed light on the etiology of PDAC and mechanisms of drug resistance in PDAC in this study.

## INTRODUCTION

Pancreatic cancer remains one of the most lethal of malignancies and a major health burden [1]. The average survival time after diagnosis with PDAC is usually less than 6 months [2]. In contrast to the steady increase in survival for most cancers, advances have been slow for pancreatic cancers, for which the 5-year relative survival is currently 8%. These low rates are partly because more than one-half of cases are diagnosed at a distant stage, for which 5-year survival is only 2% [3]. The low survival rate is attributed to several factors, of which perhaps the most important is the late stage at which most patients are diagnosed. Most patients with pancreatic cancer are asymptomatic until the disease develops to an advanced stage [4].

The lack of specific symptoms at early tumor stages and the paucity of biomarkers contribute to the poor diagnosis of PDAC. Several molecular markers have been applied for early prediction of pancreatic tumor by now [5–8]. For example, Carbohydrate antigen 19-9 (CA19-9) has a 79–81% sensitivity and 82–90% specificity for diagnosis [9]. The combination of CA19-9 and CA125 may improve sensitivity because the concentration of CA125 was raised in about 20% of CA19-9-negative cases [4, 10]. But CA19-9 is not sufficiently sensitive and specific to consistently differentiate early cancer from benign disease [11]. To improve prognosis, a screening biomarker for early diagnosis of pancreatic cancer is in urgent need. In this study, we focused on the difference of genes expression between PDAC and normal pancreas aiming at finding some potential biomarkers.

Another impetus for this study comes from the necessary to establish the etiology of PDAC. Recently, the development of pancreatic cancer has been attributable to the overexpression of several oncogenes such as KRAS [12], HIF-1α [13], MYB [14], SOX9 [15] and VEGF [16], inactivation tumor suppressor genes such as TP53 [17], or the deregulation of various signaling pathway (Hedgehog [18] and PI3K/Akt [19]). Better understanding of the pathogenesis of this disease contributes to more effective approaches to prevent PDAC.

Moreover, the deregulation of some certain genes or signaling pathways influence the effectiveness of chemotherapy and response to adjuvant chemotherapy, for example GATA6 regulates epithelial- mesenchymal transition (EMT) pathway and could be a marker of response to adjuvant chemotherapy with 5-fluorouracil (5-FU) in pancreatic cancer [20]. Knowing this in advance will influence the selection of chemotherapeutics as a result.

In this study, we intended to screen out significantly different expression genes that are replicated across microarrays datasets of PDAC and research their relationships with the prognosis of PDAC patients. We searched the public databases of expression profiling by array with the keywords’ pancreatic ductal adenocarcinoma’ and screen out 4 series of gene expression microarray data of primary PDAC tissues and the corresponding non-tumorous pancreas tissues from patients directly after excluded inappropriate studies. We identified the common significantly differentially expressed genes of these studies and selected out 3 novel candidate cancer genes in pancreatic cancer which we have proved the intimate connection between their expression levels and the prognosis of PDAC.in this study, we also demonstrated that focal adhesion also called cell–matrix adhesion and extracellular matrix were top significantly regulated pathways in PDAC. We also revealed protein-protein interactions networks of significantly differentially expressed genes that participate in the two pathways or genes functionally enriched.

## RESULTS

### Filtrating of eligible studies

We aimed at comparing gene expression profile of matched pairs of pancreatic tumor and adjacent non-tumor tissues. We searched the databases of expression profiling by array on GEO (https://www.ncbi.nlm.nih.gov/geo/) with the keywords’ pancreatic ductal adenocarcinoma’ and limited the Organism to be ‘Homo sapiens’. We got 90 items in search results, and all these studies have been carefully reviewed. The study selection flow chart is shown in Supplementary Figure 1. Initially we eliminated the studies on PDAC cellines or xenografts and other irrelevant studies. Then we excluded 24 studies which compared samples of pancreatic ductal adenocarcinoma tumor with normal pancreas tissues or non-tumor tissue from unpaired patients, 3 studies compared on PADC without chemoradiation or other neoadjuvant treatments with PADC after neoadjuvant treatments, 2 studies on peripheral blood samples and 1 study only analyzed tumor tissues. After all these scrutiny, we screen out 5 series of gene expression microarray data. We used GEO2R to calculate the distributions of value data for their samples and box plots were made to view the distributions graphically. By analyzing the boxplots, we find the boxplots of GSE39751 were not median-centered which indicative that the data may be not normalized and cross-comparable, so GSE39751 was excluded from candidates for further analysis since part of its microarray data did not meet the quality controls. Finally, we identified 4 series of gene expression microarray data as our analyzing subject and the main features of these 4 studies were shown in Table 1.

**Table 1:** The main features of 4 selected studies of gene expression microarray data

GEO Datasets	Platform	Samples in total	regions	Submission date	Citation(s) on
GSE18670	GPL570	24	Belgium	21-Oct-09	BMC Cancer 2012 Nov 16;12:527. PMID:23157946 [21]
GSE15471	GPL570	78	Romania	31-Mar-09	Hepatogastroenterology 2008 Nov-Dec;55(88):2016-27. PMID:19260470 [22]
GSE28735	GPL6244	90	USA	20-Apr-11	Clin Cancer Res 2013 Sep 15;19(18):4983-93. PMID: 23918603 [23] PLoS One 2012;7(2):e31507. PMID: 22363658 [24]
GSE58561	GPL14550	15	Norway	17-Jun-14	PLoS One 2014 Aug 22;9(8):e103873. PMID: 25148029 [25]

### Identification of significantly differentially expressed genes

**Figure 1:**
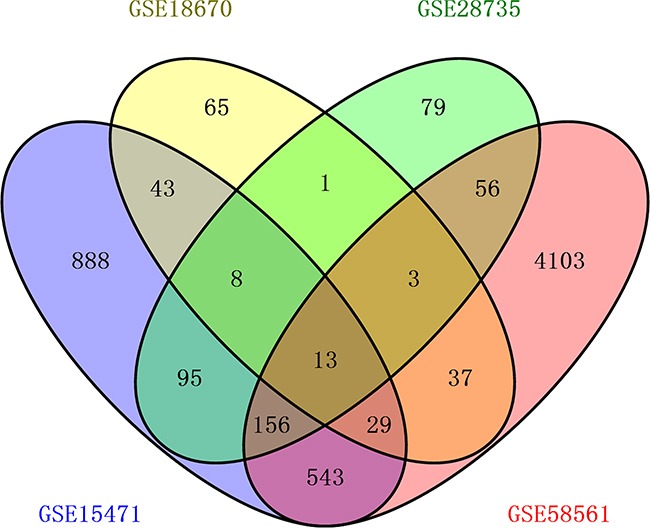
Venn diagram for 4 gene expression microarray data (“GSE15471”, “GSE18670”, “GSE28735” and “GSE58561”) Each oval represents a study. Numbers in each overlapped area means numbers of differently expression genes in each area. The brown intersection in the middle represents genes which is significantly differentially expressed in four microarray databases consistently.

After we selected “GSE18670” [21], “GSE15471” [22], “GSE28735” [23, 24] and “GSE58561” [25] for research, we used the online tool GEO2R provided by the GEO database officially to analyze values of gene expressions. All the microarray data had been normalized using the RMA algorithm. We compared the microarray gene-expression profiles of each matched pairs of pancreatic tumor and adjacent non-tumor tissues. We set |log2 (RMA signal intensity fold change) |≥1 and p value >0.05 as cut off to identified the significantly differentially expressed genes (DEGs). Then we obtained DEGs that were significantly upregulated or downregulated in each study above respectively. The consistently upregulated or downregulated genes were identified using Venn analysis and a Venn's diagram made by Venny2.1 was shown in Figure 1. 13 DEGs (MYOF, SLC6A6, S100P, HK2, IFI44L, OSBPL3, IGF2BP3, PDK4, IL1R2, ERO1A, EGLN3, PLAC8 and ACSL5) were found consistently significantly differentially expressed in four microarray databases. 12 of these DEGs (MYOF, SLC6A6, S100P, HK2, IFI44L, OSBPL3, IGF2BP3, IL1R2, ERO1A, EGLN3, PLAC8 and ACSL5) are consistently overexpressed in PDAC, while PDK4 is downregulated. To validate sequencing results obtained by four microarray databases, the expression of 13 DEGs genes have been verified by quantitative real time RT-PCR (qRT-PCR) in tumor samples and their matched non-tumor samples from 137 PDAC patients.

### ERO1A, OSBPL3 and IFI44L are potential marker for PDAC

**Figure 2:**
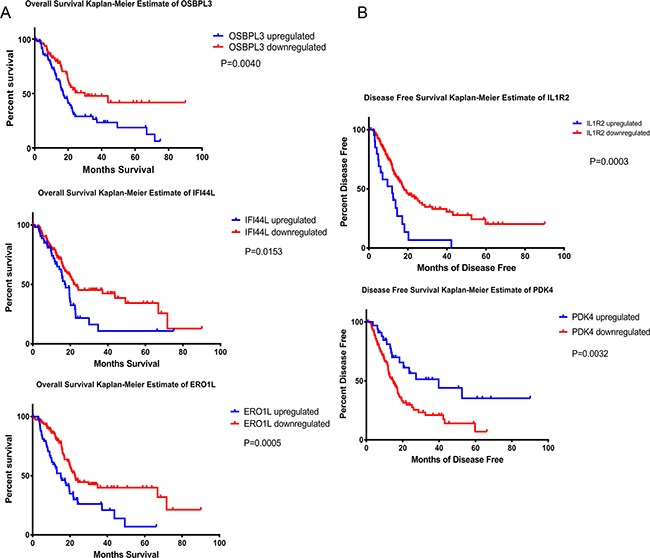
Kaplan-Meier analysis of overall survival, disease-free survival and median month's survival Patients were categorized according to DGEs expression levels. *P < 0.05, **P < 0.01, ***p<0.001.

**Figure 3:**
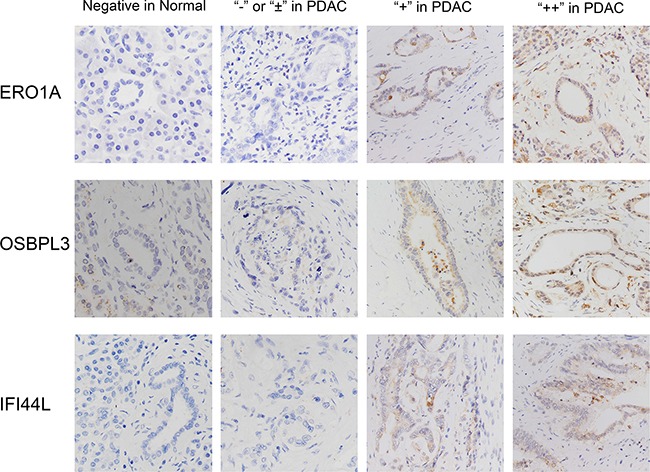
Characterization of ERO1A, OSBPL3 and IFI44L protein expression in human PDAC tissues and paired adjacent non-tumor tissues by immunohistochemistry staining and classified as strong expression (++), moderate expression (+), weak positive or negative expression (± or -) Original magnification: 200×.

**Figure 4:**
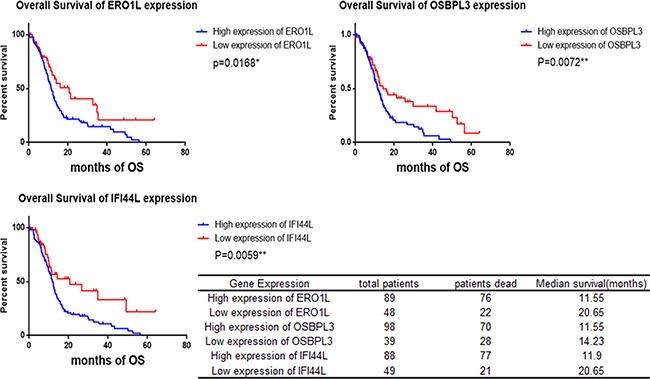
Survival analysis of PDAC patients by Kaplan-Meier plots and log-rank tests Patients were categorized by high and low expression of ERO1A, OSBPL3 and IFI44L respectively based on IHC staining scores. *p<0.05, **p<0.01.

As far as we concerned, 5 genes (ACSL5, PDK4, OSBPL3, IFI44L, and SLC6A6) have not been reported associated with PDAC. Whether the expression of IL1R2 and ERO1A are relevant clinical prognosis have not been reported yet. We next evaluated the clinical significance of these seven genes above in PDAC. The TCGA database which provides 177 PDAC patients’ mRNA expression data and clinical follow-up survey was used to estimate overall survival (OS), disease free survival (DFS) and median month's survival of each gene. Comparison of Survival Curves was carefully made and p values were calculated by Log-rank (Mantel-Cox) test. As shown in Plots of their Kaplan–Meier estimator (Figure 2A), upregulation of ERO1A (P=0.0005), OSBPL3 (p= 0.0153) and IFI44L (P=0.0040) were significantly correlated with shorter overall survival and shorter median survival time (Figure 2B), which means patients with upregulation of ERO1A, OSBPL3 and IFI44L have worse clinical prognosis than other PDAC patients who without (Table 2). Furthermore, survival analysis demonstrated that expressions of PDK4 and IL1R2 may not influence on the patients’ overall survival, whereas downregulation of PDK4 and upregulation of IL1R2 revealed IL1R2 significantly correlated with short Disease Free Survival and earlier relapsed (Figure 2C). However, even if ACSL5 and SLC6A6are also dysregulated in PDAC, there was no significant association of ACSL5 and SLC6A6 expression with clinical prognosis. Either 4 series of gene expression microarray databases or the TCGA database only revealed us the levels of DEGs’ mRNA expression. Therefore we intended to verify the results through the method of clinicopathology. 137 PDAC tissues and matched non-tumor tissue specimens were stained with anti-human antibodies of ERO1A, OSBPL3 and IFI44L respectively (Figure 3). As shown in Figure 3, ERO1A, OSBPL3 and IFI44L are negatively or weak expressed in normal pancreas tissues, and in most PDAC samples they are strongly or moderately expressed. The expression was scored as depicted in Methods and patients were categorized by high and low expression of these 3 genes based on IHC staining scores respectivel2 (Table 3). We found that elevated ERO1A, OSBPL3 and IFI44L protein expression in tumor tissues was significantly associated with late T stage which represent depth of tumor invasion and late TNM stage. As well as worse stages, Survival analysis of ERO1A (p=0.0168), OSBPL3 (p=0.0072) and IFI44L (p=0.0059) by Kaplan-Meier plots and log-rank tests revealed statistical significance between high expression group and low expression group (Figure 4). Patients with EROIL, OSBPL3 and IFI44L overexpression suffer significantly shorter median survival time. Elevated ERO1A, OSBPL3 and IFI44L protein expression in tumor tissues may cause patients’ median survival time 18.83%-44.07% (ERO1A:44.07%, OSBPL3: 18.83%and IFI44L, 42.37%) shorter than patients who without.

**Table 2:** Overall survival (OS), disease free survival (DFS) and median month's survival of ERO1A, OSBPL3 and IFI44L

	Overall Survival Kaplan-Meier Estimate			
	total cases	cases deceased	median months survival	Logrank Test P-Value
ERO1A upregulated	59	41	15.11	p=0.0004894
ERO1A downregulated	118	51	22.7	
OSBPL3 upregulated	96	61	17.02	p= 0.00397
OSBPL3 downregulated	81	31	29.99	
IFI44L upregulated	55	34	17.48	p= 0.0155
IFI44L downregulated	122	58	21.88	
	**Disease Free Survival Kaplan-Meier Estimate**			
	**total cases**	**cases relapsed**	**median months disease free**	**Logrank Test P-Value**
ERO1A upregulated	45	34	11.93	p=0.0005923
ERO1A downregulated	93	47	20.37	
OSBPL3 upregulated	71	52	13.67	p= 0.00121
OSBPL3 downregulated	67	29	25.89	
IFI44L upregulated	40	25	13.04	p= 0.0814
IFI44L downregulated	98	56	17.28	

**Table 3:** Correlation between ERO1A, OSBPL3 and IFI44L expression and clinicopathological characteristics of PDAC patients (n=137). *P<0.05,**P<0.01,***p<0.001

Variables	Number of cases	ERO1A immunostaining		P value	OSBPL3 immunostaining		P value	IFI44L immunostaining		P value
		“++or+” n=89	“±or-” n=48		“++or+” n=98	“±or-” n=39		“++or+” n=88	“±or-” n=49	
Gender										
male	76	48	28	0.621	53	23	0.603	46	30	0.312
female	61	41	20		42	19		45	16	
Age(years)										
<60	45	25	20	0.106	31	14	0.631	34	11	0.053
≥60	92	64	28		67	25		54	38	
Tumor size(cm)										
<5	56	55	26	0.386	52	29	0.022*	55	26	0.281
≥5	81	34	22		46	10		33	23	
Differentiation										
Poorly, undifferentiated (G3+G4)	39	24	15	0.596	27	12	0.706	20	19	0.046*
Well, moderately (G1+G2)	98	65	33		71	27		68	20	
T stage										
T1+T2	32	13	19	0.001**	28	4	0.022*	15	17	0.019*
T3+T4	105	76	29		70	35		73	32	
Lymphnode metastasis										
Negative	45	30	15	0.77	28	17	0.091	29	16	0.971
Positive	92	59	33		70	22		59	33	
Distant metastasis										
Negative	134	87	47	0.95	96	38	0.85	87	47	0.259
Positive	3	2	1		2	1		1	2	
TNM stage										
I+II	43	35	8	0.006**	22	21	0.00 035***	35	8	0.005**
III+IV	94	54	40		76	18		53	41	

### Pathway analysis of differentially expressed genes

**Figure 5:**
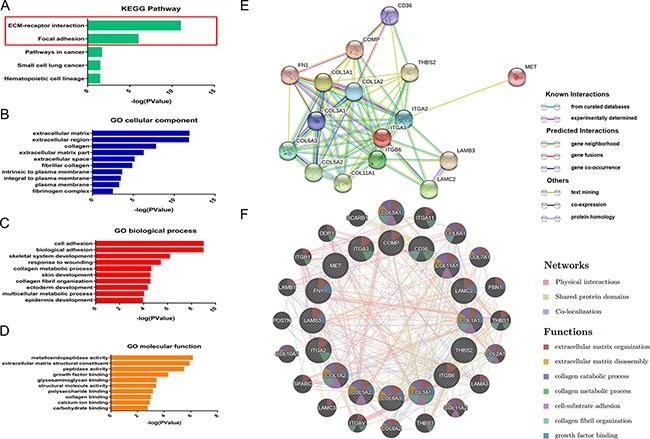
Pathway analysis of 4 microarray databases selected (GSE15471, GSE18670, GSE28735 and GSE58561) Figure 5A: KEGG pathway analyses of genes significantly differentially expressed in at least 3 of microarray databases. The most significant pathways were ECM-receptor interaction and focal adhesion; Figure 5B: The top dysregulated cellular components in PDAC is the cluster of extracellular matrix (ECM); Figure 5C: the top significant GO biological process enrichments included cell adhesion, collagen metabolic process and collagen fibril organization. Figure 5D: GO enrichment analysis of molecular function are mostly associated to extracellular matrix metabolism and extracellular matrix structural organization. Figure 5E: sixteen DEGs involved in ECM-receptor interaction and focal adhesion pathways and their interactions between each other. Figure 5F: Functionally enrichment of genes participate in pathways of ECM-receptor interaction and focal adhesion.

Furthermore, for the purpose of identifying significant pathways associated with the DEGs between PDAC tissues with normal, we ulteriorly studied 4 microarray databases previously mentioned (GSE15471, GSE18670, GSE28735 and GSE58561). DAVID Bioinformatics Resources 6.7 was used for Gene Ontology enrichment analyzing the DEGs which were significantly differentially expressed in at least 3 of microarray databases selected (GSE15471, GSE18670, GSE28735 and GSE58561). KEGG pathway analyses showed that the most significant pathways were ECM-receptor interaction and focal adhesion (Figure 5A); the top significant GO biological process enrichments included cell adhesion, collagen metabolic process and collagen fibril organization (Figure 5C). Moreover, GO enrichment analysis shows that the top significantly differentially expressed cellular components and molecular function are mostly associated to extracellular matrix (ECM) and extracellular matrix structural constituent (Figure 5B & D).

In addition, integrated PPI (protein-protein interaction) networks were more conducive to finding the crucial genes involved in the process of pancreatic ductal adenocarcinoma. Aiming at visualizing the gene lists and interactions, sixteen DEGs involved in KEGG pathway and their interactions between each other were revealed intuitively in a protein-protein interactions network with STRING 10 (Figure 5E). These sixteen genes functionally enriched with another twenty genes which regulate ECM-receptor interaction and focal adhesion pathways through physical interactions or other methods were categorized according to its functions by GeneMANIA (Figure 5F). Networks of DEGs and results of pathway analysis demonstrate that focal adhesion also called cell–matrix adhesion and extracellular matrix were top significantly regulated pathways in PDAC.

## DISCUSSION

In the present study, we determined the feasibility of three genes (ERO1A, OSBPL3 and IFI44L) working as potential biomarkers in pancreatic ductal adenocarcinoma (PDAC). In our study, we have revealed that these three genes (ERO1A, OSBPL3 and IFI44L) are significantly upregulated in PDAC and the expression level of these three genes are negative correlation with the overall survivals of patients with PDAC. This conclusion is carefully made after studies the databases of expression profiling by array on GEO and TCGA database and we carefully confirmed the conclusion with samples from our own patients.

ERO1A is an oxidizing enzyme that exists in the endoplasmic reticulum and is induced under hypoxia [26]. The oxireductase endoplasmic reticulum oxidoreductin 1-α ERO1-α was strongly upregulated by hypoxia and independently by hypoglycemia, two known accompaniments of tumors. In natural human tumors, ERO1A mRNA was specifically induced in hypoxic microenvironments coinciding with that of upregulated VEGF expression. [27]. And it has been found that ERO1-α is overexpressed in a variety of tumor types [26, 28]. Latest studies have shown that overexpression of Endoplasmic Reticulum Oxidoreductin 1-α (ERO1A) is associated with poor prognosis of breast cancer and gastric cancer [29, 30]. Likewise, hypoxic tumor microenvironment also might promote invasion and metastasis in PDAC [31]. We have illustrated that ERO1A may play critical roles in pancreatic cancer progression and represent shorter overall survival and worse clinical prognosis. The possible mechanism could be that hypoxic induction of Ero1-L alpha is the key adaptive response in the HIF-1-mediated pathway which is believed contributed to hypoxia-induced pancreatic cancer cells invasion. Zhao et al [31] and Sada et al [31]revealed that the hypoxic tumor microenvironment in PDAC might promote invasion and metastasis, besides microenvironment remodeling by hypoxic pancreatic stellate cells (PSCs) promotes cancer cell motility through alteration of extracellular matrix (ECM) fiber architecture. Nonetheless, this urged for an in depth study of the mechanism(s) driven by ERO1A in PDAC development and progression.

OSBPL3 encodes a member of the oxysterol-binding protein (OSBP) family, a group of intracellular lipid receptors. It has been reported that OSBPL3 influences cell adhesion by regulating R-Ras activity and is overexpressed in several cancers [32]. Cells lose adhesive contacts when OSBPL3 is phosphorylated, suggesting that it is subject to regulation by outside-in signals mediated by adhesion receptors. The present findings demonstrate a new function of OSBPL3 as part of the machinery that controls the actin cytoskeleton, cell polarity and cell adhesion [33]. As we known, cell adhesion is a top significantly regulated pathway in PDAC, the connection between OSBPL3 and PDAC need a further investigation. IFI44L was identified as a potential genomic biomarker in several autoimmune diseases such as systemic lupus erythematosus, rheumatoid arthritis, and Sjögren's syndrome [34–36]. IFI44L is reported upregulated in human nasopharyngeal carcinoma cells modulated through the downregulation of miR-9 [37]. In melanoma and non-melanoma skin cancer cells with CDKN2A mutated, IFI44L is also reported upregulated [38]. However these finding remains at the level of cell biology and none evident was reported that the deregulation of IFI44L may influence the progression or prognosis of cancer. We firstly provided the clinicopathology evidences which can prove PDAC patients with high IFI44L expression had a poorer overall survival.

Thus, additional studies are needed to more clearly and comprehensively articulate the molecular mechanisms of both the cause and the effects of altered expression of these three genes in the progression of PDAC.

The pancreatic cancer extracellular matrix (ECM) is composed of collagens, noncollagen glycoproteins, glycosamminoglycans, growth factors and proteoglycans as well as modulators of the cell matrix interaction such as periostin, tenascin C, SPARC (secreted protein acidic and rich in cysteine) and thrombospondin. The changes in the level of ECM proteins play a major role in invasion and migration and correlate with increased invasive potential of pancreatic cancer cells (PCCs) [39]. Furthermore, the ECM of PDAC is robust and rich in fibrillar collagens, which have been proposed to be major barrier to chemoresponse. A defining characteristic of PDAC is an intense fibrotic response that promotes tumor cell invasion and chemoresistance [40]. We explored the correlation between 3 DEGs (ERO1A, OSBPL3 and IFI44L) upregulation and clinical characteristics and prognosis of PDAC patients. In addition, two of these three DEGs (ERO1A and OSBPL3) affect the deposition of extracellular matrix and cell–matrix adhesion. We demonstrated that cell–matrix adhesion and extracellular matrix were top significantly regulated pathways in PDAC and revealed the protein-protein interaction of genes involved. These findings should encourage deeper investigation into the mechanism underlying extracellular network and 3 DEGs (ERO1A, OSBPL3 and IFI44L).

## MATERIALS AND METHODS

### Patients and tissue specimens

We obtained Primary PDAC tissues and the corresponding non-tumorous pancreas tissues (at least 2 cm away from the tumor margin) from patients who have underwent curative surgery at Shanghai Jiao tong University School of Medicine Affiliated Ruijin Hospital from 2010 to 2014. They were 76 men and 61women, with a median age of 65.2 years (range: 36–79years). None of these patients had received radiotherapy or chemotherapy before their surgery. Staging has been done clinically, radiographically, and/or pathologically. All clinicopathological data were collected and pathological tumor staging was determined according to the 7th Edition of the AJCC Cancer Staging Manual [41]. Histological typing was performed by at least two expert pathologists, working independently in a double-blinded fashion. All patients were fully informed of the experimental procedures before tissue acquisition. This study was approved by the Ethics Committee of Shanghai Ruijin Hospital. The primary PDAC tissues and the corresponding non-tumorous pancreas tissue samples were collected freshly and were snap-frozen in liquid nitrogen immediately after resection and stored at −80°C for further use. The redundant tissues were also fixed by formaldehyde and embedded by paraffin to produce tissue chips for immunohistochemistry.

### Quantitative real-time PCR (qRT-PCR)

Total RNA was isolated from cells with TRIzol reagent (Invitrogen, Carlsbad, CA, USA). Next, cDNA was synthesized with reverse transcription kit (Invitrogen, Carlsbad, CA, USA) following the manufacturer's instructions, qRT-PCR was performed on the 7500 Fast Real time PCR system (Applied Biosystem) using SYBR Green agent (Applied Biosystem) to verify the expression profile by microarrays in tumor samples and their matched non-tumor samples we collected from 137 PDAC patients accepted operations in our institution. Primers used for qRT-PCR assay are listed in the Supplementary Table 1. All PCR assays were repeated three times.

### Immunohistochemistry (IHC)

PDAC tissues sections fixed by formalin and embedded by paraffin were dewaxed in xylene and rehydrated with gradient ethanol. The sections were respectively incubated with rabbit Anti-ERO1A monoclonal antibody (1:150, Abcam, USA), rabbit Anti-IFI44L polyclonal antibody (1:150, Novus, USA) or rabbit Anti-OSBPL3 polyclonal antibody (1:150, Novus, USA) at 4°C overnight, prior to which antigen retrieval was performed by boiling in 0.01 mol/L citrate buffer (pH 6.0). The immune complex was detected by a standard avidinbiotin detection system with the LSAB+ kit (Dako, USA). The nuclei were counterstained with hematoxylin. The sections were evaluated by two pathologists who were blinded to clinicopathology information. Staining score= positive cell score + staining intensity score. The percentage of positive cells was classified by four grades (percentage scores): 0 (0), <1/3 (1), 1/3-2/3 (2) and >2/3 (3). The intensity of staining was also divided into four grades (intensity scores): no staining (0), weak staining (1), moderate staining (2) and strong staining (3). The overall scores 0, 1-2, 3-4, and 5-6 were defined as negative (−), weak in PDAC (±), moderate in PDAC (+), and strong in PDAC (++) respectively.

### Data source

Expression profile by microarrays was obtained from National Center for Biotechnology Information Gene Expression Omnibus (NCBI GEO, http://www.ncbi.nlm.nih.gov/geo/) which is regarded as a most authoritative public functional genomics data repository by now [42, 43]. All mRNA expression profiling data series involved in this study were available to public. In order to identify genes that are differentially expressed in PDAC, we used an interactive web tool GEO2R which performs comparisons on original submitter-supplied processed data tables using the GEOquery and limma R packages from the Bioconductor project to compare two or more groups of Samples in a GEO Series.

The Cancer Genome Atlas (TCGA) is a community resource project and data are made available rapidly after generation for community research use and a PDAC database from TCGA named Pancreatic Adenocarcinoma (TCGA, Provisional) provides most complete clinical information, follow-ups and mRNA expressions data of 177 samples from 185 patients diagnosed as pancreatic adenocarcinoma. They were 102 male and 83 female, aged from 35 to 88 years old. The cBioPortal for Cancer Genomics (http://www.cbioportal.org) which provides visualization, analysis and download of large-scale cancer genomics data sets was used as a tool to analyze Pancreatic Adenocarcinoma (TCGA, Provisional) in this study [44, 45].

### Selection of eligible studies

We searched the databases of expression profiling by array on GEO with the keywords’ pancreatic ductal adenocarcinoma’ and limited the Organism to be ‘Homo sapiens’. We carefully reviewed all these studies have been and eliminated ineligible studies with the following exclusion criteria: 1) studies on PDAC cellines or xenografts and other irrelevant studies; 2) studies which compared samples of pancreatic ductal adenocarcinoma tumor with normal pancreas tissues or non-tumor tissue from unpaired patients; 3) studies compared on PADC without chemoradiation or other neoadjuvant treatments with PADC after neoadjuvant treatments; 4) studies on peripheral blood samples or only analyzed tumor tissues; 5)studies which were not median-centered which indicative that the data may be not normalized and cross-comparable.

### Pathway analysis

In order to study pathways related to pancreatic ductal adenocarcinoma and evaluate the potential functions of genes regulated on the cellular level, we chose The DAVID Bioinformatics Resources 6.7(short for The Database for Annotation, Visualization and Integrated Discovery https://david.ncifcrf.gov/) which now provides a comprehensive set of functional annotation tools for investigators to understand biological meaning behind large list of genes was used to perform KEGG pathway analysis and Gene Ontology (GO) functional enrichment analysis [46, 47]. Furthermore, we identified enriched biological themes and discover enriched functional-related gene groups by DAVID. In DAVID annotation system, EASE Score, a modified Fisher Exact P-Value, is adopted to measure the gene-enrichment in annotation terms. It ranges from 0 to 1. The smaller it is, the more enriched, Fisher Exact P-Value = 0 represents perfect enrichment. The threshold of EASE Score we used is 0.1 as default.

Besides, many functional partnerships and interactions that occur between proteins are at the core of cellular processing and their systematic characterization helps to provide context in molecular systems biology. Here, the STRING database (http://string-db.org) which can provide a critical assessment and integration of protein–protein interactions, including direct (physical) as well as indirect (functional) associations was used in our study [48]. It helps to visualize the gene list contained genes involved in KEGG pathway and interactions between them in a protein-protein interaction network.

In this study, GeneMANIA [49] which searches many large, publicly available biological datasets was used to help finding and categorizing related genes, including physical interactions, sharing protein domains and Co-localization. Data sources that GeneMANIA relying on consists GEO, BioGRID (http://thebiogrid.org/), PFAM (http://pfam.sanger.ac.uk/), Pathway Commons (http://www.pathwaycommons.org/) and some other reliable and famous databases.

### Algorithm and Statistical analysis

Due to uncontrollable factors, values of the same gene on different arrays vary significantly. Thus, the raw intensity values have been background corrected and normalized by Robust Multi-array Average (RMA) which is an algorithm used to create an expression matrix from Affymetrix data.

The distribution of the IHC scoring results for each protein was analyzed by the Fisher's exact test. The postoperative survival of patients with PDAC was analyzed by the Kaplan-Meier estimator and tested by the log rank. P values < 0.05 were considered statistically significant. Statistical analysis was performed using IBM SPSS Statistics Version 19 software. The relationship between the expression level of ERO1A, OSBPL3 and IFI44L and clinicopathological parameters was examined by the Pearson χ2 test or Fisher's exact tests. The differences between the two groups were calculated by the Student's t test. The significance level was set at P < 0.05.

## SUPPLEMENTARY MATERIALS FIGURE AND TABLE




