# Supplementary material for: Integrated expression profiles analysis reveals novel predictive biomarker in pancreatic ductal adenocarcinoma
Source: Oncotarget. 2017 Mar 31;8(32):52571–83. doi: 10.18632/oncotarget.16732 (PMC5581051; doi:10.18632/oncotarget.16732)
Supplement: Supplementary file 1 [file oncotarget-08-52571-s001.pdf]

# Integrated expression profiles analysis reveals novel predictive biomarker in pancreatic ductal adenocarcinoma

## SUPPLEMENTARY MATERIALS

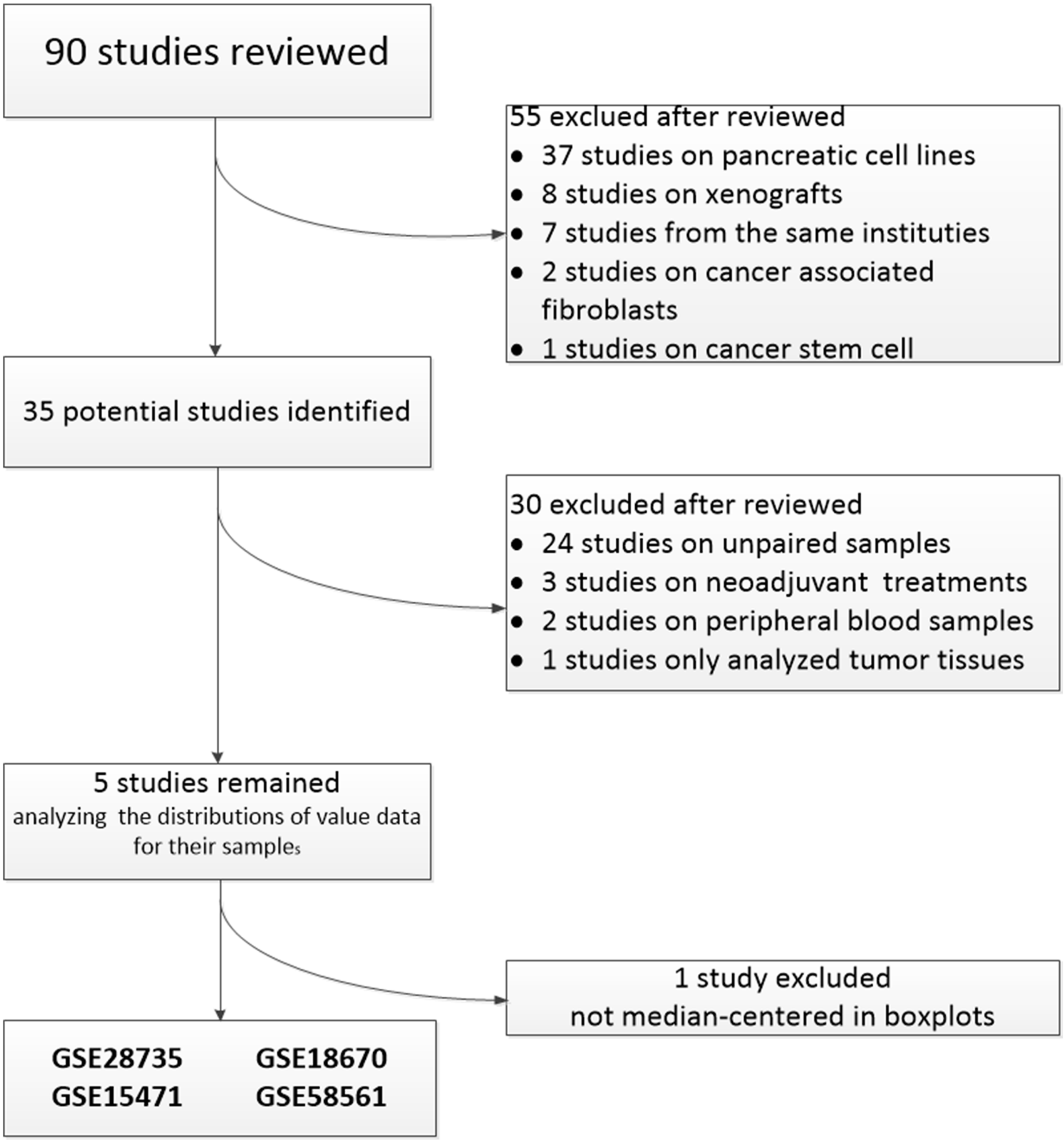


**Supplementary Figure 1: Selection of eligible studies following exclusion criteria mentioned in Methods.**

**Supplementary Table 1: Primers used for qRT-PCR assay in this article.**

**See Supplementary File**
